# Three-Dimensional Reconstruction and Virtual Simulation of Patient-Specific Anatomy for Procedural Planning in Thoracoscopic Segmentectomy: A Systematic Review and Meta-Analysis

**DOI:** 10.1093/ejcts/ezaf283

**Published:** 2025-08-26

**Authors:** Hongbo He, Chengyuan Yu, Yichen Yang, Jos G Maessen, Peyman Sardari Nia

**Affiliations:** Department of Cardiothoracic Surgery, Cardiovascular Research Institute Maastricht (CARIM), Maastricht University, 6229 ER Maastricht, The Netherlands; Department of Cardiothoracic Surgery, Cardiovascular Research Institute Maastricht (CARIM), Maastricht University, 6229 ER Maastricht, The Netherlands; Department of Cardiothoracic Surgery, Cardiovascular Research Institute Maastricht (CARIM), Maastricht University, 6229 ER Maastricht, The Netherlands; Department of Cardiothoracic Surgery, Cardiovascular Research Institute Maastricht (CARIM), Maastricht University, 6229 ER Maastricht, The Netherlands; Department of Cardiothoracic Surgery, Heart and Vascular Centre, Maastricht University Medical Centre, 6229 HX Maastricht, The Netherlands; Department of Cardiothoracic Surgery, Cardiovascular Research Institute Maastricht (CARIM), Maastricht University, 6229 ER Maastricht, The Netherlands; Department of Cardiothoracic Surgery, Heart and Vascular Centre, Maastricht University Medical Centre, 6229 HX Maastricht, The Netherlands

**Keywords:** three-dimensional reconstruction, segmentectomy, procedural planning, systematic review, meta-analysis

## Abstract

**Background:**

Three-dimensional reconstruction of patient-specific anatomy and virtual simulation for procedural planning in thoracoscopic segmentectomy could theoretically improve the clinical outcomes. Therefore, the aim of this study was to evaluate the contemporary evidence to test this hypothesis.

**Methods:**

Four databases (PubMed, Embase, Cochrane Library, Web of Science) were searched for articles published before October 12, 2024. Intraoperative parameters (operative time, intraoperative blood loss, the number of lymph node resections, inadequate margin) and postoperative parameters (postoperative complications, total chest drainage, chest tube duration, postoperative hospital stay) were the comparative end-points.

**Results:**

A total of 10 articles were included in this study. 772 patients were in the three-dimensional (3D) reconstruction group and 652 patients were in the non-three-dimensional (non-3D) reconstruction group. The procedural planning with 3D reconstruction and simulation reduced the probability of inadequate surgical margins (Odds ratio [OR] = 0.09; 95% confidence interval (CI)  = 0.02-0.50; *P* = .006) and postoperative complication rates (OR = 0.53; 95% CI = 0.38-0.74; *P* < .001). In the subgroup analysis, 3D reconstruction reduced the operative time (Mean difference [MD] = −10.85 min; 95% CI = −15.39 to −6.02, *P* < .001) and intraoperative blood loss (MD = −5.41 ml; 95% CI = −9.87 to -0.94, *P* = .020) in complex segmentectomies. As for the number of lymph node resections, chest tube duration, total chest drainage, and postoperative hospital stay, the 2 groups were similar with no statistically significant difference.

**Conclusions:**

Patient-specific 3D reconstruction and simulation for procedural planning in segmentectomy may help reduce the probability of inadequate surgical margins and complications. In complex segmental resections, it may shorten the operative time and reduce intraoperative blood loss.

## Graphical abstract

**Figure:**
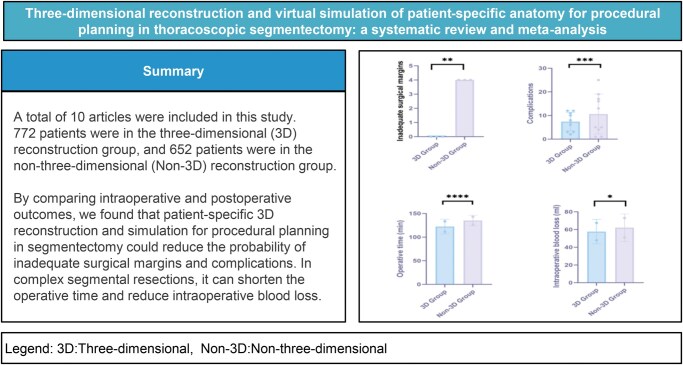

## INTRODUCTION

Lung Cancer remains the leading cause of cancer mortality in the World.^1^ Low-dose computed tomography (LDCT) improves detection of early-stage non-small-cell lung cancer.^2^ Currently, surgery remains the most effective treatment for early-stage lung cancer. The JCOG0802 and CALGB/Alliance 140503 large clinical phase 3 trials showed no significant difference in postoperative complications and mortality between segmentectomy and lobectomy.^3^^,^^4^ Consequently, thoracic surgeons are increasingly using segmental lung resections to treat lung nodules less than 2 cm in diameter. However, segmental lung resection requires clear understanding of spatial relation of the tumour location, the segment, bronchus, artery and vein.^5^

With advances in imaging technology, it is now possible to convert a 2D chest CT into a 3D image. Three-dimensional (3D) reconstruction clearly shows the spatial relation of blood vessels and bronchial tree, vascular branching patterns, anatomical variations, and helps to precisely locate the lesion in relation to the target segment.^6^^,^^7^ Controversy remains regarding the benefits of procedural planning and simulation via three-dimensional image reconstruction. In recent years, several retrospective studies have demonstrated shorter operative times, less intraoperative blood loss, adequate surgical margin distances, fewer postoperative complications, and reduced postoperative drainage in patients in the three-dimensional reconstruction group.^8–10^ A prospective study by Chen et al^11^showed that there was no statistical difference in perioperative outcomes between patients in the three-dimensional reconstruction group (3D group) and those in the non-three-dimensional reconstruction group (Non-3D group). Moreover, current studies exhibit certain limitations. Most are single-centre studies with small sample sizes, and their conclusions are susceptible to interference and have limited generalizability. Potential biases and confounding factors, such as differences in surgeon experience and variations in the technical level of 3D reconstruction software, are difficult to eliminate completely, and these factors cause inconsistencies in the results. So we performed a meta-analysis of all eligible studies to investigate the clinical value of three-dimensional reconstruction for lung segmental resection.

## METHODS

This meta-analysis followed the Preferred Reporting Items for Systematic Reviews and Meta-Analyses (PRISMA) guideline of 2020.^12^ PROSPERO registration number is CRD42024611761.

### Literature search strategy

This study searched 4 databases (PubMed, Embase, Cochrane Library, and Web of Science) for articles published before October 12, 2024. We used the MeSH terms “Imaging, Three-Dimensional” and “Thoracic Surgery, Video-Assisted” as follows. In addition, we manually reviewed the reference lists within the included articles to further identify potentially relevant studies (other sources). Specific search strategies can be seen in the **Table S1**.

### Inclusion criteria and exclusion criteria

**Figure 1.**
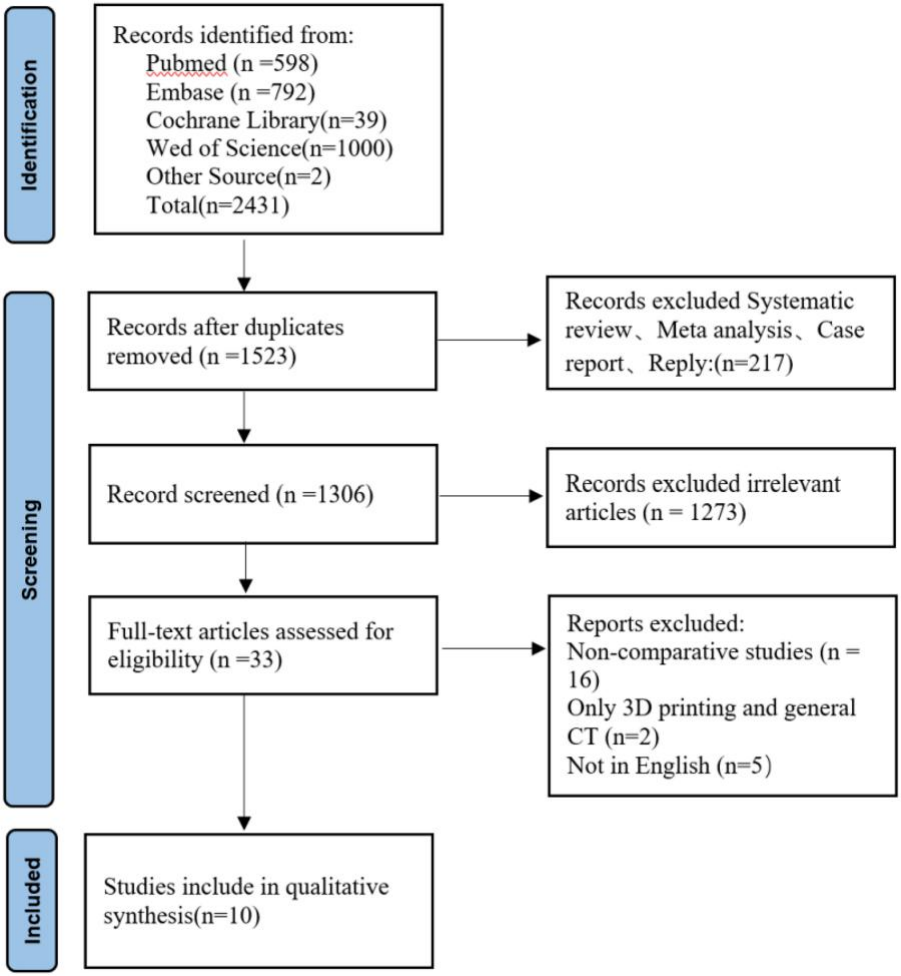
PRISMA Search Flow Diagram

In this study, articles were screened according to PICOS with the following screening criteria (**Figure 1**).

#### Inclusion criteria

1. P (Population): Patients undergoing thoracoscopic segmental lung resection (including benign lung lesions and lung malignancies).
2. I and C (Intervention and comparison): Comparison of perioperative outcomes in patients undergoing segmental lung resection by whether or not they receive preoperative simulation with three-dimensional reconstructed images.
3. O (Outcomes): Intraoperative indicators (operative time, intraoperative blood loss, the number of lymph nodes, inadequate margin) and postoperative indicators (postoperative complications, total chest drainage, chest tube duration, postoperative hospital stay).
4. S (study): Randomized controlled trials and cohort studies.

#### Exclusion criteria

1. Inappropriate article types: non-comparative studies, systematic review, meta-analysis, reply and case reports.
2. Duplicate and irrelevant literature.
3. Only 3D printing and general CT were compared in the study.
4. Not in English.

### Data extraction

We constructed a standardized table to extract the data. Two authors (Hongbo He and Chengyuan Yu) independently extracted the data. Any disagreement was resolved by discussion until consensus was reached by consulting a third author (Peyman Sardari Nia). We used EndNote software for literature management and screening. A total of 2431 articles were included. In the first round of screening, duplicate articles were excluded (*n* = 908). In the second round of screening, systematic review, meta-analysis, case report, and reply (*n* = 217) were excluded. The third round of screening excluded irrelevant articles (*n* = 1273). The fourth round of screening excluded non-comparative studies (*n* = 16), only 3D printing and general CT (*n* = 2), and those not in English (*n* = 5). General information included the first author, country, publication date, study duration, number of patients, gender, average age, complex segmentectomies, study type, and study outcomes. The meta-analysis outcomes included operative time, intraoperative blood loss, number of lymph node resections, inadequate margin, postoperative complications, total chest drainage, chest tube duration, and postoperative hospital stay. For quantitative data without mean and standard deviation, alternative methods were used to estimate the data based on median and interquartile range. We calculated the mean using the method proposed by Luo et al.^13^ We calculated the standard deviation using the method proposed by Wan et al.^14^ In the studies where propensity matching and non-propensity matching were used, we chose the data from the propensity matching analysis. Simple segmentectomy is the left upper division, lingula, superior segment (S6), and basal segment. Other segmentectomies are classified as complex segmentectomies.^15^^,^^16^ Inadequate margins are defined as positive margins found during intraoperative frozen section diagnosis.

### Quality assessment

We assessed cohort studies using The Newcastle-Ottawa Scale (NOS). The scale is divided into 3 dimensions: selection, comparability, and outcome. On a scale of 0-9, ≥7 was considered high quality, 5-6 was considered moderate quality, and ≤4 was recognized as low quality. Randomized controlled trials were assessed using the Jadad scale. The scale contained randomization (0-2 points), study blinding (0-2points), and withdrawal (0-1 point) categories. With ≥3 considered high quality, ≤2 was considered low quality.

### Statistical analysis

We performed statistical analysis using STATA 17.0 and Review Manager Version 5.3 (Software Update, Cochrane Collaboration, UK, Oxford). Odds ratios (OR) were used for dichotomous variables. Mean differences (MDs) were used for continuous variables. All effect estimates are reported with the 95% confidence interval (CI). The X^2^-based Q-statistic test and I^2^ test were used to assess the statistical heterogeneity. I^2^ < 50% and *P*-value > .10 suggested acceptable heterogeneity and then a fixed-effect model was applied. Otherwise, significant heterogeneity existed and a random-effect model was used. Sensitivity analyses were performed by removing each study to observe the stability of the results. Publication bias using funnel plots and the Egger test. *P*-values <.05 were defined as significant.

## RESULTS

### General characteristics

A total of 10 articles were included in this study, 9 were cohort studies and 1 a randomized controlled trial. Nine studies were conducted in China, 1 in Japan. The total number of patients was 1424, of which 772 were in the 3D group and 652 were in the non-3D group (**Table 1**). All the studies were found to be of moderate to high quality (**Table 2**). Considering the high cost of 3D printing and the lack of large-sample prospective trials to validate clinical benefits, therefore, 3D printed models were not included.

**Table 1.** Summary of the Baseline Characteristics of the Included Studies

First author (year)	Nation	Period	Total (3D/non-3D)	Sex (M/F)	Age (3D/non-3D)	Complex segmentectomies (3D/non-3D)	Type of trial	Outcomes
Xu^21^ 2019.11	China	2017.07-2018.11	133(96/37)	43/80	50.4 ± 11.7/53.3 ± 12.3	85/31	Cohort study	1 2 3 6 7 8
Wang^8^ 2022.09	China	2021.01-2022.03	97(42/55)	28/69	55.3 ± 13.2/59.4 ± 13.9	42/55	Cohort study	1 3 4 6 7 8
Wang^31^ 2022.09	China	2013.12-2021.06	139(85/54)	46/93	49.6 ± 8.2/49.1 ± 5.3	85/54	Cohort study	1 3 5 7
Wu^32^ 2021.04	China	2020.01-2020.09	110(55/55)	43/67	52.9 ± 10.4/53.1 ± 11.9	NG	Cohort study	1 2 3 4 5 7 8
Qiu^28^ 2020.08	China	2017.04-2019.05	267(131/136)	77/190	54.4 ± 8.7/54.4 ± 9.6	79/76	Cohort study	1 3 5 6 8
Xue^9^ 2018.12	China	2016.05-2017.02	68(36/32)	21/47	53.0 ± 11.1/51.6 ± 10.5	26/22	Cohort study	1 3 5 7 8
Chen^11^ 2024.07	China	2019.06-2023.11	191(95/96)	55/83	53.9 ± 12.9/54.7 ± 11.0	66/68	Randomized control trial	1 2 3 5 6 7 8
He^10^ 2024.09	China	2016.01-2022.02	265(148/117)	118/137	56.0 ± 10.7/54.5 ± 11.8	NG	Cohort study	1 2 3 5 7 8
Sekine^16^ 2019.09	Japan	2014.10-2018.04	62(31/31)	30/32	70.3 + 9.4/70.3 + 9.4	NG	Cohort study	1 3 7
Liu^33^ 2019.12	China	2017.10-2018.08	92(53/39)	32/60	62.1 ± 9.7/60.6 ± 11.2	22/23	Cohort study	1 3 5 7 8

Abbreviations: F, Female; M, male; NG, not given; 3D, three-dimensional group; Non-3D, non-three-dimensional group; 1, Operation time; 2, the number of lymph node resections; 3, intraoperative blood loss; 4, inadequate margin; 5, chest tube duration; 6, total chest drainage; 7, postoperative complications; 8, postoperative hospital stay.

**Table 2.** Methodological Quality Assessments of the Included Studies

**Study**	Selection				Comparability	Outcome			Total score
Cohort (NOS)	Exposed cohort	Nonexposed cohort	Ascertain of exposure	Outcome of interest		Assessment of outcome	Length of follow-up	Adequacy of follow-up	
Guobing Xu	*	*	*	–	**	*	–	–	6
Xinyu Wang	*	*	*	–	**	*	*	–	7
Mingbo Wang	*	*	*	–	**	*	–	–	6
Xianning Wu	*	*	*	–	**	*	*	–	7
Bin Qiu	*	*	*	–	*	*	–	–	5
Liang Xue	*	*	*	–	**	*	*	*	8
Hao He	*	*	*	–	**	*	–	–	6
Yasuo Sekine	*	*	*	–	**	*	–	–	6
Xiaojun Liu	*	*	*	–	**	*	–	–	6
Randomized control trial (Jadad)		Random sequence production			Blinding method			Withdrawal	
Kai Chen		**			–			*	3

Abbreviation: NOS, Newcastle-Ottawa Scale. Asterisks denotes quality assessment scores: * represents 1 point, and ** represents 2 points.

### Operative time

**Figure 2.**
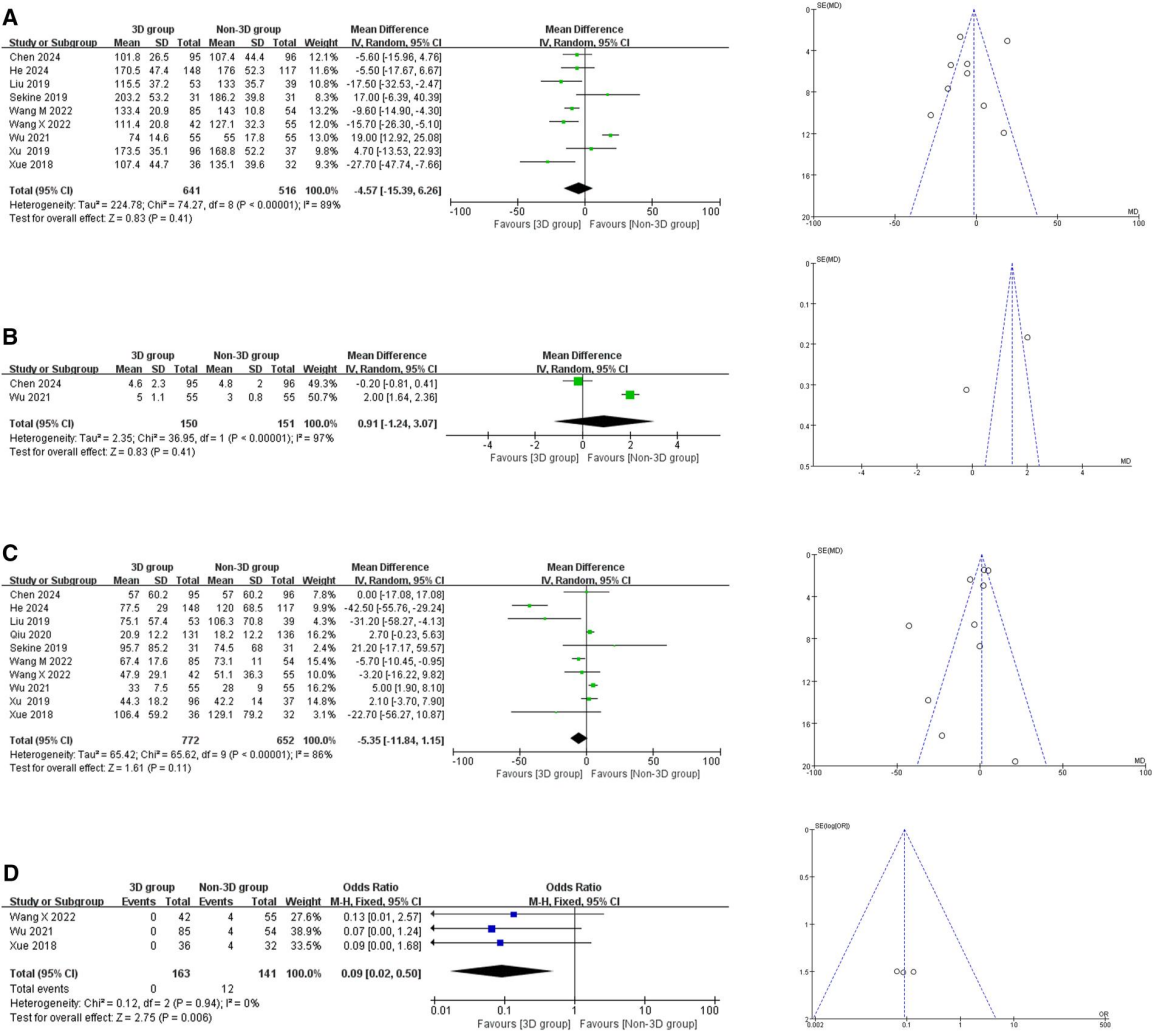
Forest Plot and Funnel Plot of Intraoperative Indicators. (A) Operating time; (B) the number of lymphnode resections; (C) intraoperative blood loss; (D) inadequate margin. Abbreviations: IV, inverse variance; M-H, Mantel-Haenszel; CI, confidence interval

A total of 9 articles and 1157 patients were included. After the heterogeneity test, I^2^ = 89%, Q test *P* < .001, suggesting high heterogeneity and therefore a random effect model was used. Meta-analysis results showed that operative time is not associated with procedural planning with 3D reconstruction and simulation (MD = −4.57 min, 95% CI = −15.39 to 6.26; *P* = .41, **Figure 2A**).

### The number of lymph node resections

A total of 2 articles and 301 patients were included. After heterogeneity test, I^2^ = 97%, Q test *P* < .001, suggesting high heterogeneity, a random effect model was used. Meta-analysis results showed that the number of lymph node resections are not associated with procedural planning with 3D reconstruction and simulation (MD = 0.91, 95% CI = −1.24 to 3.07; *P* = .41, **Figure 2B**).

### Intraoperative blood loss

A total of 10 articles and 1424 patients were included. After the heterogeneity test, I^2^ = 86%, Q test *P* < .001, suggesting high heterogeneity in this study, a random-effects model was used. Meta-analysis results showed that intraoperative blood loss is not associated with preoperative 3D reconstruction and simulation (MD = −5.35 mL, 95% CI = −11.84 to 1.15; *P* = 0.110, **Figure 2C**).

### Inadequate margin

A total of 3 articles with 294 patients were included. After testing for heterogeneity, I^2^ = 0%, Q-test *P* = .94, suggesting low heterogeneity in this study, a fixed effect size was used to combine the effects. Meta-analysis results showed that compared with the non-3D group, patients in the 3D group had a reduced probability of inadequate surgical margins, and there was a statistically significant difference between the 2 groups (OR = 0.09, 95% CI = 0.02 to 0.50; *P* = .006, **Figure 2D**).

### Chest tube duration

**Figure 3.**
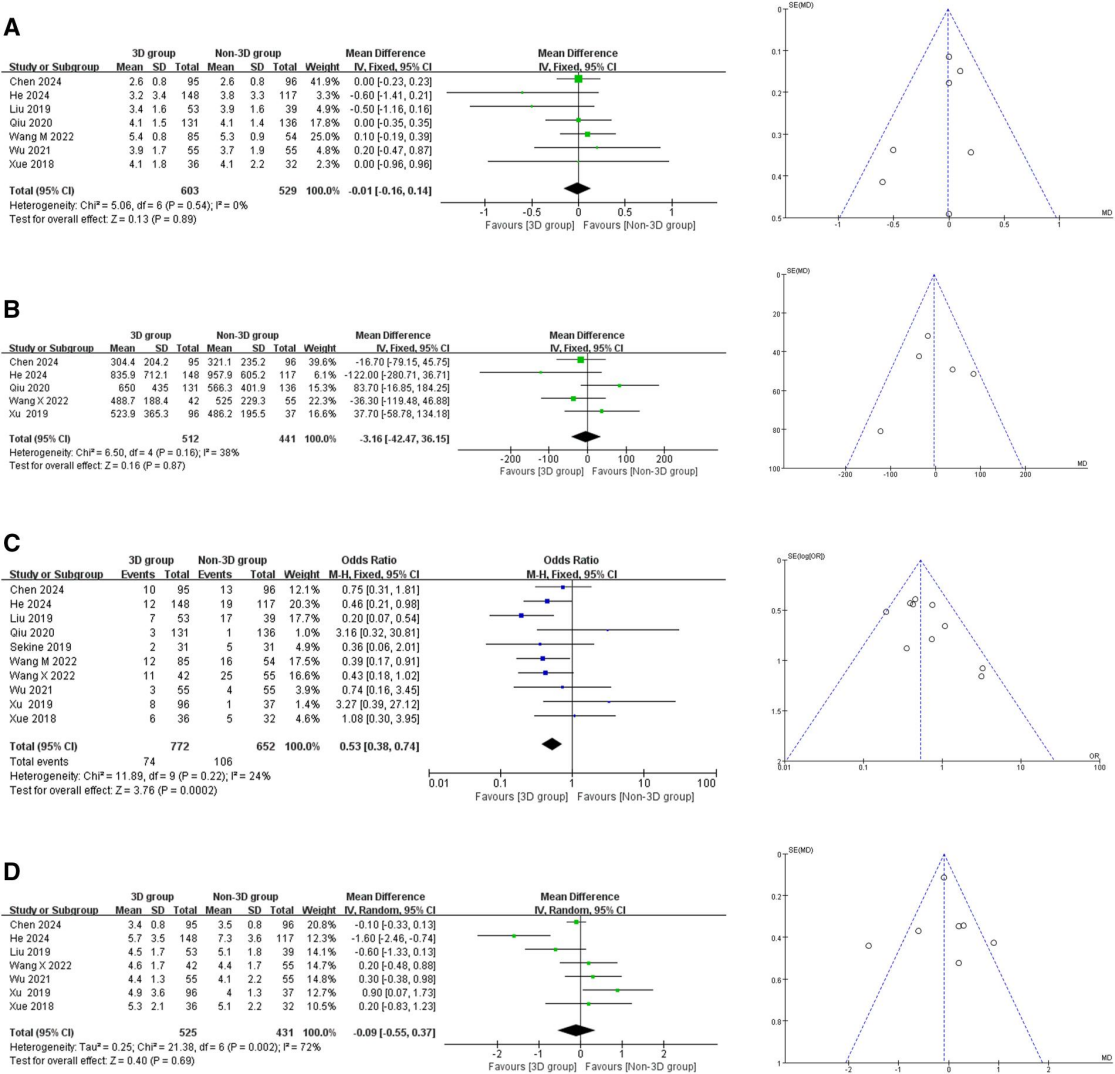
Forest Plot and Funnel Plot of Postoperative Indicators. (A) Chest tube duration; (B) total chest drainage; (C) postoperative complications; (D) postoperative hospital stay. Abbreviations; IV, inverse variance; M-H, Mantel-Haenszel; CI, confidence interval

A total of 7 articles and 1132 patients were included. After the test of heterogeneity, I^2^ = 0%, Q test *P* = .54, suggesting low heterogeneity in this study, a fixed-effects model was used. Meta-analysis results showed that chest tube duration is not associated with preoperative 3D reconstruction and simulation (MD = −0.01 days, 95% CI = −0.16 to 0.14; *P* = .89, **Figure 3A**).

### Total chest drainage

A total of 5 articles and 953 patients were included. After the heterogeneity test, I^2^ = 38%, Q test *P* = .160, suggesting moderate heterogeneity in this study, a fixed effect model was used. Meta-analysis results showed that the total chest drainage is not associated with preoperative 3D reconstruction and simulation (MD = −3.16 mL, 95% CI = −42.47 to 36.15; *P* = .87, **Figure 3B**).

### Postoperative complications

A total of 10 articles and 1424 patients were included. After the heterogeneity test, I^2^ = 24%, Q test *P* = .22, suggesting low heterogeneity in this study, a fixed-effects model was used. Meta-analysis results showed that patients in the 3D group had fewer complications compared to the non-3D group, with a statistically significant difference between the 2 groups (OR = 0.53, 95% CI = 0.38 to 0.74; *P* < .001, **Figure 3C**).

### Postoperative hospital stay

A total of 7 articles and 956 patients were included. After heterogeneity test, I^2^ = 72%, Q test *P* = .002, suggesting high heterogeneity in this study, a random-effects model was used. Meta-analysis results showed that postoperative hospital stay was not associated with preoperative 3D reconstruction and simulation (MD = −0.09 days, 95% CI = −0.55 to 0.37; *P* = .69, **Figure 3D**).

### Sensitivity analysis

Since there are few articles involving the number of lymph node resections and inadequate margins, we did not choose to perform a sensitivity analysis. We performed sensitivity analyses on other results using fixed-effect models and random-effect models, respectively. It was found that the exclusion of each study did not influence the results (**Figures S1 and S2**).

### Publication bias

We performed the Egger’s test for operative time (**Figure S3A**), intraoperative blood loss (**Figure S3B**), and postoperative hospital stay (**Figure S3C**) and found no publication bias.

### Subgroup analysis and meta-regression

There was high heterogeneity due to operative time, intraoperative blood loss, and postoperative hospital stay. We performed the subgroup analysis for these factors. Subgroup analyses were conducted according to year of publication (≥ 2020 vs < 2020), sample size (>130 vs < 130), age (>55 vs < 55), method (propensity score matching vs non-propensity score matching), and surgical complexity (simple and complex vs complex) (**Table 3**).

**Table 3.** Subgroup Analysis and Meta-Regression

Factor	Studies	Study heterogeneity		Effect model	*P* value	Pooled OR/MD (95%CI)	Meta regression
		I² (%)	*P* (heterogeneity)			(3D group vs non-3D group)	*P* value
**Operative time**							
Overall	9	89%	<0.001	Random	0.41	−4.57 (−15.39, 6.26)	
Year of publication							0.78
≥2020	5	93%	<0.001	Random	0.65	−3.23 (−17.27, 10.82)	
<2020	4	74%	0.009	Random	0.48	−6.67 (−25.14, 11.80)	
Sample size							0.74
>130	4	0%	0.47	Random	<0.001	−7.63 (−11.90, −3.35)	
<130	5	93%	<0.001	Random	0.65	−4.87 (−25.89, 16.15)	
Age							0.65
>55	4	61%	0.050	Random	0.160	−8.17 (−19.66, 3.32)	
<55	5	93%	<0.001	Random	0.72	−2.92 (−18.72, 12.87)	
Method							0.005
PSM	2	0%	0.87	Fixed	<0.001	18.87 (12.98, 24.76)	
Non-PSM	7	32%	0.180	Fixed	<0.001	−9.97 (−13.74, −6.21)	
surgical complexity							0.23
Simple and complex	7	87%	<0.001	Random	0.78	−1.97 (−15.61, 11.66)	
Complex	2	2%	0.31	Random	<0.001	−10.85 (−15.67, −6.02)	
**Intraoperative blood loss**							
Overall	10	86%	<0.001	Random	0.24	1.08 (−0.71, 2.87)	
Year of publication							0.95
≥2020	6	91%	<0.001	Random	0.160	−5.65 (−13.47, 2.17)	
<2020	4	65%	0.040	Random	0.47	−7.47 (−27.82, 12.87)	
Sample size							0.56
>130	5	92%	<0.001	Random	0.140	−7.30 (−16.93, 2.33)	
<130	5	65%	0.020	Random	0.54	−4.09 (−17.11, 9.92)	
Age							0.190
>55	4	87%	<0.001	Random	0.22	−16.49 (−43.09, 10.11)	
<55	6	69%	0.007	Random	0.67	0.88 (−3.18, 4.94)	
Method							0.060
PSM	2	0%	0.41	Random	0.001	5.10 (2.02, 8.19)	
Non-PSM	8	87%	<0.001	Random	0.040	−8.99 (−17.35, −0.63)	
surgical complexity							0.64
Simple and complex	8	87%	<0.001	Random	0.150	−5.91 (−13.89, 2.06)	
Complex	2	0%	0.72	Random	0.020	−5.41 (−9.87, −0.94)	
**Postoperative hospital stay**							
Overall	7	72%	0.002	Random	0.69	−0.09 (−0.55, 0.37)	
Year of publication							0.65
≥2020	4	78%	0.003	Random	0.45	−0.24 (−0.85, 0.38)	
<2020	3	72%	0.003	Random	0.76	0.15 (−0.78, 1.08)	
Sample size							0.58
>130	3	88%	<0.001	Random	0.65	−0.25 (−1.32, 0.82)	
<130	4	22%	0.28	Random	0.93	0.02 (−0.41, 0.44)	
Age							0.120
>55	3	81%	0.006	Random	0.21	−0.64 (−1.63, 0.35)	
<55	4	51%	0.110	Random	0.34	0.22 (−0.23, 0.66)	

Abbreviations: Non-PSM, non-propensity score matching; PSM, propensity score matching.

For operative time, studies using propensity score matching (PSM) showed a significant increase in operative time in the 3D group (MD = 18.87 min, 95% CI: 12.98 to 24.76, *P* < .001), whereas studies that did not use PSM showed a significant decrease in operative time in the 3D group (MD = −9.97 min, 95% CI: −13.74 to −6.21, *P* < .001). In sample sizes > 130, a significant reduction in operative time was observed in the 3D group (MD = −7.63 min, 95% CI: −11.90 to −3.35, *P* < .001). In the complex surgery group, a significant reduction in operative time was observed in the 3D group (MD = −10.85 min, 95% CI: −15.67 to −6.02, *P* < .001). Meta-regression analysis showed that the method was the source of heterogeneity in operative time (*P* = .005) (**Table 3**).

For intraoperative blood loss, studies using PSM showed a significant increase in blood loss in the 3D group (MD = 5.10 mL, 95% CI: 2.02 to 8.19, *P* = .001), whereas studies without PSM showed a significant decrease in blood loss in the 3D group (MD = −8.99 mL, 95% CI: −17.35 to −0.63, *P* = .040), and a subgroup of complex surgeries showed a significant reduction in blood loss in the 3D group (MD = −5.41 mL, 95% CI: −9.87 to −0.94, *P* = 0.020). Meta-regression analyses revealed no sources of heterogeneity (**Table 3**).

For postoperative hospital stay, in all subgroup analyses, no significant differences in postoperative hospital stay were found between the 3D groups and non-3D group. Meta-regression analyses revealed no sources of heterogeneity (**Table 3**).

## DISCUSSION

Pulmonary segmentectomy, as a precise and minimally invasive surgical method, is increasingly used in early-stage lung cancer. Considering the complexity of the procedure, it is difficult to assess the variability of the patient’s lung anatomy with routine 2D CT. Therefore, 3D reconstruction techniques are beginning to be widely used in thoracic surgery. The advantage of 3D reconstruction is that it can convert 2D images of pulmonary arteries, pulmonary veins, and bronchi into 3D images of the vascular tree and bronchial tree, clearly displaying the spatial relation of blood vessels and bronchial tree, effectively evaluating the branching patterns of blood vessels and detecting anatomical variations.^5^ This meta-analysis aimed to systematically assess the clinical value of preoperative planning in 3D reconstruction.

For intraoperative outcomes, we found that patients in the 3D group had more adequate margin distances. Both groups were similar in terms of the number of lymph nodes dissected with no statistically significant difference. In subgroup analyses that included 2 studies of patients who underwent only complex segmental lung resections, we found that patients in the 3D group had shorter operative time and less intraoperative blood loss. More than one-third of p-T1N0M0 NSCLC tumours extended beyond 1 segment, irrespective of size.^17^ Thus, for segmentectomy of a single lung segment, which is usually performed along the segmental vein with the aid of an inflation-deflation line, does not always provide an adequate surgical margin. However, preoperative simulation using 3D image reconstruction and intraoperative extended segmental resection allows for adequate surgical margins to be obtained.^18^ The reason that there was no difference in operative time and intraoperative blood loss in the overall group is that simple lung segmentectomy surgeons relied on previous experience and familiarity with anatomy, making the advantage of preoperative 3D reconstruction not obvious. For complex lung segmentectomy with more vascular variants and complex operations, the surgeon’s assessment of the abnormal pulmonary vasculature by preoperative 3D imaging reduces the probability of intraoperative vascular injury, which in turn reduces the operative time and intraoperative blood loss. Several studies demonstrated that three-dimensional reconstruction is more than 95% accurate in detecting pulmonary arteries.^19^^,^^20^ The number of lymph node resections was similar in both groups, which on the one hand may be related to intraoperative lymph node sampling rather than lymph node dissection in early-stage lung cancer.^21^ On the other hand, only two articles in this study recorded the number of lymph nodes resected, and there was a high degree of heterogeneity (I^2^ = 97%) in the analysed results, which may have been biased.

For postoperative outcomes, we found that patients in the 3D group had fewer postoperative complications. This may be due to the preoperative 3D reconstructed images, which helped the surgeon plan the optimal surgical path to avoid inadvertent injury to vital airways and blood vessels, and to reduce the possibility of postoperative bleeding and postoperative airleak. Meanwhile, the shorter operating time means less anaesthesia time and surgery-related trauma for the patient, reducing the incidence of postoperative infections and other complications. In the study by Liu et al on preoperative 3D reconstruction for hepatectomy, we also observed a reduction in complications in the 3D group of patients.^22^ In terms of chest tube duration, total chest drainage, and postoperative hospital stay, both groups were similar with no statistically significant difference. In recent years, the large-scale use of thoracoscopic segmental lung resection has led to a reduction in lung tissue damage, thus greatly decreasing chest tube drainage and chest tube duration.^23^^,^^24^ At the same time, the proposal of enhanced recovery after surgery (ERAS) in thoracic surgery has also reduced the length of hospitalization of patients. This resulted in similar postoperative outcomes in the 3D group and non-3D group.

A recent meta-analysis by Xiang et al included eight studies (*n* = 989) that combined both 3D image reconstruction and 3D printing. The results of the meta-analysis showed that preoperative 3D lung simulation could significantly decrease the blood loss, operative time, conversion rate (conversion from segmentectomy to thoracotomy or lobectomy), postoperative hospital stay and total number of complications compared with non-3D procedures. The number of lymph node resections, postoperative drainage time, postoperative forced expiratory volume in the first second and total chest drainage were similar in the 2 groups.^25^

Consistent with Xiang et al, our meta-analysis similarly demonstrated significant reductions in postoperative complications, but there was no improvement in operative time, intraoperative blood loss, and postoperative hospital stay. This discrepancy may be attributed to Xiang et al’s inclusion of physical 3D printed models, which offer surgeons superior tactile feedback and spatial perception compared to 3D image reconstruction alone. This was confirmed by studies by Chen et al^26^ and Li et al^27^ who compared 2 groups of patients who underwent preoperative 3D image reconstruction and 3D printing. Patients in the 3D printing group were found to have shorter surgery times and less blood loss than those in the 3D image reconstruction group. Qiu et al conducted a questionnaire survey of 59 surgeons who had used 3D reconstruction and 3D printing models, and also confirmed that these visualization differences led to inconsistent results. 88% of surgeons agreed that 3D printing provides a better understanding of thoracic anatomy than 3D reconstruction. 50.9% of surgeons strongly agreed that 3D printing is more convenient to use during surgery than 3D reconstruction imaging. 81.4% of surgeons agreed or strongly agreed that 3D printing may help reduce potential surgical complications.^28^

Meanwhile, our study found additional benefits that were not addressed in Xiang’s study. We demonstrated that patients who underwent preoperative 3D reconstruction had significantly improved surgical margin adequacy, an important surgical outcome that was overlooked in previous study. Furthermore, our meta-analysis explicitly proposes that simple and complex pulmonary segmentectomy should be studied separately. Our subgroup analysis of complex pulmonary segmentectomy showed that even with 3D reconstruction, operative time and intraoperative blood loss were reduced. These results are highly consistent with the overall conclusions of Xiang et al and further confirm the effectiveness of 3D technology in thoracic surgical planning.

Although 3D reconstruction has significant clinical advantages in preoperative planning for pulmonary segmentectomy, its promotion still faces multiple challenges. On the one hand, although fully automated commercial 3D reconstruction platforms can quickly generate individualized models, they are difficult to popularize in some medical institutions due to their high procurement costs. On the other hand, free semi-automated reconstruction software such as 3D Slicer requires clinicians to invest more time in manual or semi-manual segmentation, but its operational process is relatively straightforward, and the detailed anatomical information obtained significantly enhances surgical safety and precision. Considering the benefits of 3D reconstruction, we believe that promoting 3D reconstruction using low-cost, open-source platforms is feasible and has practical application value in resource-constrained institutions. For 3D printing technology, high costs and a lack of low-cost alternatives mean that its current promotion in clinical settings is even more insufficient. Meanwhile, whether there is a real benefit compared to 3D image reconstruction needs to be verified in a large prospective study, so we did not include 3D printing models in our study.

In our other subgroup analyses, we observed the opposite outcomes: in PSM cohorts, the 3D group exhibited longer operative times and greater intraoperative blood loss compared to the non-3D group; conversely, in non-PSM cohorts, the 3D group showed significant reductions in both operative time and blood loss. A similar decrease in operative time was also observed in studies with larger sample sizes (more than 130 patients). It is possible that the use of PSM to exclude unmatched patient information resulted in data loss, creating heterogeneity. Kane et al^29^ and Lin et al^30^ have noted that, although PSM balances observed covariates between groups, it does so by discarding unmatched controls, resulting in information loss and reduced statistical power.

Our study also has some limitations. First, most of the included studies were retrospective cohort studies, and there was only one randomized controlled trial. In retrospective studies, patients are usually included based on available medical records, and missing information often leads to the exclusion of certain cases, resulting in biased results. Meanwhile, without preoperative adjustment for key factors such as pulmonary function, tumour stage, and comorbidities, baseline imbalances between groups can arise and potentially bias the results. Secondly, although some studies have used propensity matching to balance known covariates, retrospective designs are still not immune to selection bias. For example, surgeons preferentially employ pre-operative 3D planning for segmentectomies they anticipate to be technically demanding, resulting in a higher proportion of complex cases in the 3D group. Because most published studies have not analysed simple and complex segmentectomies separately, this non-random, selective allocation may bias the observed outcomes. In addition, some surgeons may have more experience and sophistication in thoracoscopic pulmonary segmentectomy. These surgeon-level confounding factors may positively affect outcomes (lower margin positivity and fewer complications) independently of the 3D technique itself. Future randomized controlled trials should stratify randomization by surgical complexity and try to maintain consistency in surgical team experience to minimize the impact of such confounding factors. Third, most of the articles included in our study did not follow-up the patients, and we could not obtain the 5-year survival and recurrence rates of the patients. Fourth, 9 of the articles included in this study were conducted in China, and one was conducted in Japan, resulting in a study population consisting mainly of Asian patients. Due to possible differences in surgical practices and perioperative management between Eastern and Western centres, the generalizability of the results of this study to non-Asian populations may be limited. Finally, only 2 articles in our study compared complex segmentectomy and the number of articles was too low.

## CONCLUSION

In conclusion, preoperative simulation using 3D image reconstruction before surgeon’s lung segmental resection is beneficial. It may help obtain adequate surgical margins and reduce complications. And in complex lung segmental surgery, it may shorten the operation time and reduce blood loss.

## Supplementary Material

ezaf283_Supplementary_Data

## Data Availability

Detailed data from this article can be found in the tables and figures of the article, and the corresponding author can be contacted for further needs.
