# Supplementary material for: Three-Dimensional Reconstruction and Virtual Simulation of Patient-Specific Anatomy for Procedural Planning in Thoracoscopic Segmentectomy: A Systematic Review and Meta-Analysis
Source: Eur J Cardiothorac Surg. 2025 Aug 26;67(9):ezaf283. doi: 10.1093/ejcts/ezaf283 (PMC12453671; doi:10.1093/ejcts/ezaf283)
Supplement: ezaf283_Supplementary_Data [file ezaf283_supplementary_data.zip › Supplementary Figures S1 to S3.doc]

Supplementary Figure S1 for sensitivity analysis (Fixed effects model): A: Operative time B: Intraoperative blood loss C: Chest tube duration D: Total chest drainage E: Postoperative complications F: Postoperative hospital stay

Supplementary Figure S2 for sensitivity analysis (Random effects model): A: Operative time B: Intraoperative blood loss C: Chest tube duration D: Total chest drainage E: Postoperative complications F: Postoperative hospital stay

Supplementary Figure S3 for publication bias: A Operative time B Intraoperative blood loss C Postoperative hospital stay
