# Supplementary material for: Three-Dimensional Reconstruction and Virtual Simulation of Patient-Specific Anatomy for Procedural Planning in Thoracoscopic Segmentectomy: A Systematic Review and Meta-Analysis
Source: Eur J Cardiothorac Surg. 2025 Aug 26;67(9):ezaf283. doi: 10.1093/ejcts/ezaf283 (PMC12453671; doi:10.1093/ejcts/ezaf283)
Supplement: ezaf283_Supplementary_Data [file ezaf283_supplementary_data.zip › Supplementary Table S1.doc]

Supplementary Table S1| Search strategy

**Pubmed (**N**=**598**)**
1 "Imaging, Three-Dimensional"[Mesh]
2 ((((((((((((((((((((((((((((((((((((((((((Imaging, Three Dimensional[Title/Abstract]) OR (Three-Dimensional Imaging[Title/Abstract])) OR (Three-Dimensional Imagings[Title/Abstract])) OR (3-D Imaging[Title/Abstract])) OR (3 D Imaging[Title/Abstract])) OR (3-D Imagings[Title/Abstract])) OR (Imaging, 3-D[Title/Abstract])) OR (Imagings, 3-D[Title/Abstract])) OR (Imaging, Three-Dimensional, Computer Assisted[Title/Abstract])) OR (Computer-Assisted Three-Dimensional Imaging[Title/Abstract])) OR (Computer Assisted Three Dimensional Imaging[Title/Abstract])) OR (Computer-Assisted Three-Dimensional Imagings[Title/Abstract])) OR (Imaging, Computer-Assisted Three-Dimensional[Title/Abstract])) OR (Imagings, Computer-Assisted Three-Dimensional[Title/Abstract])) OR (Three-Dimensional Imaging, Computer-Assisted[Title/Abstract])) OR (Three-Dimensional Imagings, Computer-Assisted[Title/Abstract])) OR (Computer-Generated 3D Imaging[Title/Abstract])) OR (3D Imaging, Computer-Generated[Title/Abstract])) OR (3D Imagings, Computer-Generated[Title/Abstract])) OR (Computer Generated 3D Imaging[Title/Abstract])) OR (Computer-Generated 3D Imagings[Title/Abstract])) OR (Imaging, Computer-Generated 3D[Title/Abstract])) OR (Imagings, Computer-Generated 3D[Title/Abstract])) OR (Three-Dimensional Imaging, Computer Generated[Title/Abstract])) OR (Three Dimensional Imaging, Computer Generated[Title/Abstract])) OR (3-D Image[Title/Abstract])) OR (3 D Image[Title/Abstract])) OR (3-D Images[Title/Abstract])) OR (Image, 3-D[Title/Abstract])) OR (Images, 3-D[Title/Abstract])) OR (Three-Dimensional Image[Title/Abstract])) OR (Images, Three-Dimensional[Title/Abstract])) OR (Image, Three-Dimensional[Title/Abstract])) OR (Three Dimensional Image[Title/Abstract])) OR (Three-Dimensional Images[Title/Abstract])) OR (three Dimensional[Title/Abstract])) OR (Three Dimension[Title/Abstract])) OR (3D Dimensional[Title/Abstract])) OR (3D Dimension[Title/Abstract])) OR (3-D dimensional[Title/Abstract])) OR (3-D demension[Title/Abstract]))))
3 1 OR 2
4 "Thoracic Surgery, Video-Assisted"[Mesh]
5(("Thoracic Surgery, Video-Assisted"[Mesh]) OR ((((((((((((((((((((((Surgeries, Video-Assisted Thoracic[Title/Abstract]) OR (Surgery, Video-Assisted Thoracic[Title/Abstract])) OR (Thoracic Surgeries, Video-Assisted[Title/Abstract])) OR (Thoracic Surgery, Video Assisted[Title/Abstract])) OR (Video-Assisted Thoracic Surgeries[Title/Abstract])) OR (Surgery, Thoracic, Video-Assisted[Title/Abstract])) OR (VATS[Title/Abstract])) OR (VATSs[Title/Abstract])) OR (Video-Assisted Thoracic Surgery[Title/Abstract])) OR (Video Assisted Thoracic Surgery[Title/Abstract])) OR (Video-Assisted Thoracoscopic Surgery[Title/Abstract])) OR (Surgeries, Video-Assisted Thoracoscopic[Title/Abstract])) OR (Surgery, Video-Assisted Thoracoscopic[Title/Abstract])) OR (Thoracoscopic Surgeries, Video-Assisted[Title/Abstract])) OR (Thoracoscopic Surgery, Video-Assisted[Title/Abstract])) OR (Video-Assisted Thoracoscopic Surgeries[Title/Abstract])) OR (Video Assisted Thoracoscopic Surgery[Title/Abstract])) OR (segmentectomy[Title/Abstract])) OR (Lung segment resection[Title/Abstract])) OR (Pulmonary segmentectomy[Title/Abstract])) OR (Segmental resection[Title/Abstract])) OR (Anatomic segmentectomy[Title/Abstract])))
6 4 OR 5
7 3 AND 6

**Embase(**N**=**792**)**
1'three-dimensional imaging'/exp
2'imaging, three dimensional':ti,ab,kw OR 'three-dimensional imaging':ti,ab,kw OR 'three-dimensional imagings':ti,ab,kw OR '3-d imaging':ti,ab,kw OR '3 d imaging':ti,ab,kw OR '3-d imagings':ti,ab,kw OR 'imaging, 3-d':ti,ab,kw OR 'imagings, 3-d':ti,ab,kw OR 'imaging, three-dimensional, computer assisted':ti,ab,kw OR 'computer-assisted three-dimensional imaging':ti,ab,kw OR 'computer assisted three dimensional imaging':ti,ab,kw OR 'computer-assisted three-dimensional imagings':ti,ab,kw OR 'imaging, computer-assisted three-dimensional':ti,ab,kw OR 'imagings, computer-assisted three-dimensional':ti,ab,kw OR 'three-dimensional imaging, computer-assisted':ti,ab,kw OR 'three-dimensional imagings, computer-assisted':ti,ab,kw OR 'computer-generated 3d imaging':ti,ab,kw OR '3d imaging, computer-generated':ti,ab,kw OR '3d imagings, computer-generated':ti,ab,kw OR 'computer generated 3d imaging':ti,ab,kw OR 'computer-generated 3d imagings':ti,ab,kw OR 'imaging, computer-generated 3d':ti,ab,kw OR 'imagings, computer-generated 3d':ti,ab,kw OR 'three-dimensional imaging, computer generated':ti,ab,kw OR 'three dimensional imaging, computer generated':ti,ab,kw OR '3-d image':ti,ab,kw OR '3 d image':ti,ab,kw OR '3-d images':ti,ab,kw OR 'image, 3-d':ti,ab,kw OR 'images, 3-d':ti,ab,kw OR 'three-dimensional image':ti,ab,kw OR 'images, three-dimensional':ti,ab,kw OR 'image, three-dimensional':ti,ab,kw OR 'three dimensional image':ti,ab,kw OR 'three-dimensional images':ti,ab,kw OR 'three dimensional':ti,ab,kw OR 'three dimension':ti,ab,kw OR '3d dimensional':ti,ab,kw OR '3d dimension':ti,ab,kw OR '3-d dimensional':ti,ab,kw OR '3-d demension':ti,ab,kw
3 #1 OR #2
4'video assisted thoracoscopic surgery'/exp
5'thoracic surgery, video-assisted':ti,ab,kw OR 'surgeries, video-assisted thoracic':ti,ab,kw OR 'surgery, video-assisted thoracic':ti,ab,kw OR 'thoracic surgeries, video-assisted':ti,ab,kw OR 'thoracic surgery, video assisted':ti,ab,kw OR 'video-assisted thoracic surgeries':ti,ab,kw OR 'surgery, thoracic, video-assisted':ti,ab,kw OR vats:ti,ab,kw OR vatss:ti,ab,kw OR 'video-assisted thoracic surgery':ti,ab,kw OR 'video assisted thoracic surgery':ti,ab,kw OR 'video-assisted thoracoscopic surgery':ti,ab,kw OR 'surgeries, video-assisted thoracoscopic':ti,ab,kw OR 'surgery, video-assisted thoracoscopic':ti,ab,kw OR 'thoracoscopic surgeries, video-assisted':ti,ab,kw OR 'thoracoscopic surgery, video-assisted':ti,ab,kw OR 'video-assisted thoracoscopic surgeries':ti,ab,kw OR 'video assisted thoracoscopic surgery':ti,ab,kw OR segmentectomy:ti,ab,kw OR 'lung segment resection':ti,ab,kw OR 'pulmonary segmentectomy':ti,ab,kw OR 'segmental resection':ti,ab,kw OR 'anatomic segmentectomy':ti,ab,kw
6 #4 OR #5
7#3 AND #6

**Cochrance library (**N**=**39**)**
1 Imaging,Three-Dimensional"[Mesh]
2 (imaging, three dimensional):ti,ab,kw OR (three-dimensional imaging):ti,ab,kw OR (three-dimensional imagings):ti,ab,kw OR (imaging, three-dimensional, computer assisted):ti,ab,kw OR (computer-assisted three-dimensional imaging):ti,ab,kw OR (computer assisted three dimensional imaging):ti,ab,kw OR (computer-assisted three-dimensional imagings):ti,ab,kw OR (imaging, computer-assisted three-dimensional):ti,ab,kw OR (imagings, computer-assisted three-dimensional):ti,ab,kw OR (three-dimensional imaging, computer-assisted):ti,ab,kw OR (three-dimensional imagings, computer-assisted):ti,ab,kw OR (computer-generated 3d imaging):ti,ab,kw OR (3d imaging, computer-generated):ti,ab,kw OR (3d imagings, computer-generated):ti,ab,kw OR (computer generated 3d imaging):ti,ab,kw OR (computer-generated 3d imagings):ti,ab,kw OR (imaging, computer-generated 3d):ti,ab,kw OR (imagings, computer-generated 3d):ti,ab,kw OR (three-dimensional imaging, computer generated):ti,ab,kw OR (three dimensional imaging, computer generated):ti,ab,kw OR (three-dimensional image):ti,ab,kw OR (images, three-dimensional):ti,ab,kw OR (image, three-dimensional):ti,ab,kw OR (three dimensional image):ti,ab,kw OR (three-dimensional images):ti,ab,kw OR (three dimensional):ti,ab,kw OR (three dimension):ti,ab,kw OR (3d dimensional):ti,ab,kw OR (3d dimension):ti,ab,kw OR (3-d dimensional):ti,ab,kw OR (3-d demension):ti,ab,kw
3 1 OR 2
4 Thoracic surgery, video-assisted"[Mesh]
5 (thoracic surgery, video-assisted):ti,ab,kw OR (surgeries, video-assisted thoracic):ti,ab,kw OR (surgery, video-assisted thoracic):ti,ab,kw OR (thoracic surgeries, video-assisted):ti,ab,kw OR (thoracic surgery, video assisted):ti,ab,kw OR (video-assisted thoracic surgeries):ti,ab,kw OR (surgery, thoracic, video-assisted):ti,ab,kw OR (VATS):ti,ab,kw OR (VATSs):ti,ab,kw OR (video-assisted thoracic surgery):ti,ab,kw OR (video assisted thoracic surgery):ti,ab,kw OR (video-assisted thoracoscopic surgery):ti,ab,kw OR (surgeries, video-assisted thoracoscopic):ti,ab,kw OR (surgery, video-assisted thoracoscopic):ti,ab,kw OR (thoracoscopic surgeries, video-assisted):ti,ab,kw OR (thoracoscopic surgery, video-assisted):ti,ab,kw OR (video-assisted thoracoscopic surgeries):ti,ab,kw OR (video assisted thoracoscopic surgery):ti,ab,kw OR OR (segmentectomy):ti,ab,kw OR (lung segment resection):ti,ab,kw OR (pulmonary segmentectomy):ti,ab,kw OR (segmental resection):ti,ab,kw OR (anatomic segmentectomy):ti,ab,kw
6 4 OR 5
7 3 AND 6

**Web of Science** (N=1000)
1 TS=(imaging, three dimensional OR three-dimensional imaging OR three-dimensional imagings OR imaging, three-dimensional, computer assisted OR computer-assisted three-dimensional imaging OR computer assisted three dimensional imaging OR computer-assisted three-dimensional imagings OR imaging, computer-assisted three-dimensional OR imagings, computer-assisted three-dimensional OR three-dimensional imaging, computer-assisted OR three-dimensional imagings, computer-assisted OR computer-generated 3d imaging OR 3d imaging, computer-generated OR 3d imagings, computer-generated OR computer generated 3d imaging OR computer-generated 3d imagings OR imaging, computer-generated 3d OR imagings, computer-generated 3d OR three-dimensional imaging, computer generated OR three dimensional imaging, computer generated OR three-dimensional image OR images, three-dimensional OR image, three-dimensional OR three dimensional image OR three-dimensional images OR three dimensional OR three dimension OR 3d dimensional OR 3d dimension OR 3-d dimensional OR 3-d demension)

2 TS=(Thoracic Surgery, Video-Assiste OR Surgeries, Video-Assisted Thoracic OR Surgery, Video-Assisted Thoracic OR Thoracic Surgeries, Video-Assisted OR Thoracic Surgery, Video Assisted OR Video-Assisted Thoracic Surgeries OR Surgery, Thoracic, Video-Assisted OR VATS OR VATSs OR Video-Assisted Thoracic Surgery OR Video Assisted Thoracic Surgery OR Video-Assisted Thoracoscopic Surgery OR Surgeries, Video-Assisted Thoracoscopic OR Surgery, Video-Assisted Thoracoscopic OR Thoracoscopic Surgeries, Video-Assisted OR Thoracoscopic Surgery, Video-Assisted OR Video-Assisted Thoracoscopic Surgeries OR Video Assisted Thoracoscopic Surgery OR segmentectomy OR Lung segment resection OR Pulmonary segmentectomy OR Segmental resection OR Anatomic segmentectomy)
3 1 AND 2
